# Visualizing Cell Cycle Phase Organization and Control During Neural Lineage Elaboration

**DOI:** 10.3390/cells9092112

**Published:** 2020-09-17

**Authors:** Fatma Rabia Urun, Adrian W Moore

**Affiliations:** 1 Laboratory for Neurodiversity, RIKEN Center for Brain Science, 2-1 Hirosawa, Wako, Saitama 351-0198, Japan; fatma.urun@riken.jp; 2 Graduate School of Science and Engineering, Saitama University, Sakura-ku, Saitama 338-8570, Japan

**Keywords:** neural stem cell, intermediate precursor cell, FUCCI, cyclin dependent kinase inhibitor, Hes1, cell cycle phase, p27^KIP1^

## Abstract

In neural precursors, cell cycle regulators simultaneously control both progression through the cell cycle and the probability of a cell fate switch. Precursors act in lineages, where they transition through a series of cell types, each of which has a unique molecular identity and cellular behavior. Thus, investigating links between cell cycle and cell fate control requires simultaneous identification of precursor type and cell cycle phase, as well as an ability to read out additional regulatory factor expression or activity. We use a combined FUCCI-EdU labelling protocol to do this, and then apply it to the embryonic olfactory neural lineage, in which the spatial position of a cell correlates with its precursor identity. Using this integrated model, we find the CDKi p27^KIP1^ has different regulation relative to cell cycle phase in neural stem cells versus intermediate precursors. In addition, Hes1, which is the principle transcriptional driver of neural stem cell self-renewal, surprisingly does not regulate p27^KIP1^ in this cell type. Rather, Hes1 indirectly represses p27^KIP1^ levels in the intermediate precursor cells downstream in the lineage. Overall, the experimental model described here enables investigation of cell cycle and cell fate control linkage from a single precursor through to a lineage systems level.

## 1. Introduction

Neural stem cells (NSCs) undergo self-renewal or differentiate into intermediate precursor cells (IPCs). In turn, these IPCs have a more limited ability to divide than NSCs, and upon differentiation, they exit from the cell cycle. In both these NSC and IPC neural precursor populations, cell cycle regulators not only control progression through the cell cycle, but also simultaneously regulate the switch between proliferation and differentiation [1,2,3]. To investigate the links between control of the cell cycle and control of the self-renewal vs. differentiation switch, it is critical to develop approaches that enable detection of which precursor type is being assayed and provide a simultaneous readout of the cell cycle phase of that cell. Moreover, because the stem cell niche itself also constrains neuronal precursor cell cycle phase parameters [4,5,6], it is important to do these analyses on a lineage in situ.

Development of such methodologies will now allow closer examination of the relationship between cell cycle phase control and neurogenesis, along with investigating how NSCs and IPCs are similar or different. One example showing why this is important comes from studies of cortical neurogenesis. As embryogenesis proceeds, there is a steady lengthening of cortical neuronal precursor cell G1 phase, and a correlated increased probability of precursor exit from self-renewal. A series of experiments that manipulated G1/S phase transition regulators showed that experimental lengthening of G1 is correlated with a reduction in self-renewal; moreover, experimental shortening of G1 increased self-renewal [7,8,9,10,11]. One interpretation of these data is that lengthening G1 causes a higher probability of exit from self-renewal (possibly due to precursors having more opportunity to receive signaling cues) [3]. Yet, when cortical NSCs and IPCs are considered as separate populations, the interpretation becomes more nuanced. G1 lengthening primarily correlates with changes in the proportion of NSCs to IPCs; on the other hand, it is a specific S phase length shortening that correlates with a higher propensity to differentiate into neurons (irrespective of precursor type) [8,12]. A second example comes from analyses of Cyclin Dependent Kinase inhibitor (CDKi) activities. p27^KIP1^ is expressed in both NSC and IPC compartments [11,13,14]. Intriguingly, local variations in p27^KIP1^ activity may underlie regionalization of neuron number in the primate brain; and in a clinical setting, it is dysregulated in glioblastoma, medulloblastoma, and ependymoma [15,16,17]. Overall p27^KIP1^ gain- and loss-of-function analyses indicate that it regulates the amount of neuron production from a lineage [11,18,19,20]. Importantly however, in a study where cortical NSCs and IPCs were examined separately, disrupting p27^KIP1^ activity only altered the differentiation capacity of IPCs [20]. Is this mirrored by differences in the control over p27^KIP1^ expression in different precursor cell types? Importantly, p27^KIP1^ activity is both a function of its expression level and of the point to which a cycling cell has progressed through the cell cycle [1,21,22]; how this occurs in precursors is unknown.

Moreover, what spatial and temporal relationships exist between regulatory information input and its readout as a neural lineage proceeds? For example, it is well-described that the bHLH factor Hes1 is the principle transcriptional driver of the self-renewal program in NSCs. Hes1 is a transcriptional repressor that directly binds the promoters of the bHLH proneural genes, preventing them from achieving the high stable expression through which they initiate NSC-to-IPC fate change [23]. Several studies have shown that Hes1 also binds and represses the p27^KIP1^ promoter (*Cdkn1b* locus) [24,25,26,27], and in proliferating HeLa cells, this regulatory relationship delays the G1/S phase transition [26]. p27^KIP1^ is thus considered a direct transcriptional target of Hes1 in NSCs, such that Hes1 repression of p27^KIP1^ increases proliferation and reduces differentiation [22]. On the other hand, experiments suggest that p27^KIP1^ does not function in NSCs, but rather in IPCs [13,20]. To begin to understand what the differences in cell cycle regulatory action between precursor types are, we need to be able to assay how they are regulated in relation to the cell cycle phases of these cells.

Here, we optimize a protocol that combines detection of transgenic FUCCI(SA) cell cycle phase markers [28,29] with 5-ethynyl-2′-deoxyuridine (EdU) labelling [30] and further markers of cell identity. FUCCI (SA) is a transgenic labelling system based on cell cycle-dependent degradation of the fluorescent proteins (FPs) fused to domains of Geminin [hGem (1/110)] and Cdt1 [hCdt1 (30/120)] that cause them to undergo selective protein destruction in G1 or S/G2/M phases, respectively. Cells that are co-labelled with mVenus-hGem (1/110) and mCherry-hCdt1 (30/120) are red in G1, and switch to green in S/G2/M [28,29].

Building on pioneering studies that described the embryonic olfactory epithelium (OE) lineage as having archetypal neural lineage organization [31,32,33,34,35], we combine the in vivo FUCCI/EdU labelling protocol with this lineage. We develop this as an integrated experimental model that has exceptional clarity for detecting the relationship between cell cycle, cell types, and transitions. Then, by examining p27^KIP1^ expression across the cell cycle in different neural precursor cell types and the relationship between Hes1 activity and p27^KIP1^ expression, we illustrate how this system can be used to dissect relationship between cell cycle phase and cell fate transitions in a neuronal lineage.

## 2. Materials and Methods

### 2.1. Mouse Stocks

We used the R26p-Fucci2 mouse strain, which uses the Rosa26 promoter to drive mCherry-hCdt1(30/120) and mVenus-hGem (1/110) from a single locus [36]. These are available from RIKEN BDR, Kobe, Japan; stock #CDB0203T. *Hes1* knockout mice (*Hes1*^tm1Fgu^) [37] were a kind gift from Dr Kageyama, University of Kyoto, Kyoto, Japan. These are available from RIKEN BRC, Japan, stock #RBRC05979. All animals were maintained in the RIKEN CBS Animal Facility and handled in accordance with RIKEN and national regulations.

### 2.2. Embryo Preparation

For EdU labelling, pregnant mice were injected intraperitoneally with EdU (50 mg/kg of body weight, in ddH2O) 30 min prior to embryo isolation. We optimized embryo fixation at 1% PFA in 0.1 M Phosphate Buffer at 4 °C overnight. Embryos were further processed for embedding in Tissue-Tek O.C.T. compound (Sakura Finetek, Tokyo, Japan) by standard protocols. We took coronal cryosections at 14 μm thickness.

### 2.3. Multiprobe Staining of Sections

To bleach the endogenous FP fluorescence before staining, the sections were placed under high illumination on an LED lightbox for 30 min. Next, we used heat induced epitope retrieval using HistoVT One (Nacalai Tesque, Kyoto, Japan) for 20 min at 70 °C (this step is important to achieve high quality multimarker detection using FUCCI-EdU). Finally, the sections were blocked with 10% normal donkey serum (NDS) in PBST (0.1% Tween20 in PBS) for 1 h at room temperature.

For optimal four-color results, the primary antibody incubation requires the serial use of antibodies, each one overnight at 4 °C. We used 1% NDS in PBST for antibody dilution, and PBST for washes.

For the data shown in Figures 4–6, we included a biotin-streptavidin amplification step for p27^KIP1^ staining. The protocol was carried out as follows: on day 1, anti-p27^KIP1^ [overnight, 4 °C]; on day 2, anti-Sox2 [overnight, 4 °C]; on day 3, anti-mCherry [overnight, 4 °C]; on day 4, samples were treated with biotinylated secondary antibody to amplify the p27^KIP1^ signal [overnight, 4 °C]. Samples were then incubated with secondary antibodies for 2 h at room temperature. Finally, a Click-iT EdU reaction was performed using the Click-iT EdU Imaging Kit (#C10337 ThermoFisher, Waltham, MA USA).

For the data shown in Figures 2 and 3, we used similar overnight staining steps. In these cases, the series of antibodies used were: anti-Sox2; anti-mCherry and anti-GFP. These steps were followed by the Click-iT EdU reaction. In Figure 3 we included a 5 min treatment with DAPI (1 µg/mL in PBS) as the final staining step.

Antibodies used in this study: goat anti-Sox2 (#sc-17320, 1:300, Santa Cruz, Dallas, TX, USA), mouse anti-mCherry (#632543, 1:500, Takara Bio Clontech, Kusatsu, Shiga, Japan), mouse anti-p27Kip1 (#610241, 1:100, BD Biosciences, San Jose, CA, USA), mouse anti-TuJ1 (#MMS-435P, 1:300, Covance, San Diego, CA, USA), rat anti-GFP (#04404-84, 1:1000, Nacalai Tesque, Kyoto, Japan), mouse anti-Ascl1 (#556604, 1:100 BD Biosciences), rabbit anti-DsRed (#632496 1:1000, ClonTech), rat anti-Hes1 (#D134-3, 1:500, MBL, Tokyo, Japan), rabbit anti-Phospho-Histone H3 (#9701 1:400 Cell Signalling Technology, Tokyo, Japan); biotinylated anti-mouse (#715-065-150, 1:200, Jackson Immunoresearch, West Grove, PA, USA), Alexa Fluor 488 anti-rat (#712-545-153, 1:500, ThermoFisher), Alexa Fluor 488 anti-goat (#705-546-147, 1:500, ThermoFisher), Alexa Fluor 647 Streptavidin (#S32357 1:500, ThermoFisher), Cy3 anti-mouse (1:500, Jackson Immunoresearch, #715-166-151), DyLight 405 anti-goat (1:500, Jackson Immunoresearch, #705-475-147).

### 2.4. Imaging and Quantitation

Samples were mounted with Dako Fluorescence Mounting Medium (Dako, Carpinteria, CA, USA). Images were acquired by using an FV1200 scanning confocal microscope (Olympus, Tokyo, Japan) with a UPLSAPO40X2 objective.

For quantitation of fluorescence levels, we used single confocal sections through the nucleus. We traced the nucleus manually using ImageJ, then we measured integrated fluorescence intensity. To calculate background levels for the use of a specific primary antibody (anti-mCherry or anti-p27^KIP1^), we took a similar-sized region of interest, and for each sample measured five unstained tissue regions in the section, then used the average of these regions as the background level. Representative maximum intensity measurements are reported in Figure S2A,B. For the majority of the OE cells in vivo, the 3D organization of the tissue means we are not quantifying from the nucleus at the position of its maximum area. Therefore, we calculated the fluorescence intensity in fluorescence units (FU) as (integrated fluorescent intensity/area) [14] for comparative measurements.

Statistical analyses were carried out using Prism 6 (GraphPad, San Diego, CA, USA). The Alpha value was *p* < 0.05. In each experiment, the test and corrections for multiple testing are stated in the figure legend.

## 3. Results

### 3.1. Systematic Labelling of All Cell Cycle Phases in Each Precursor Type of an Embryonic Neuronal Lineage

**Figure 1:**
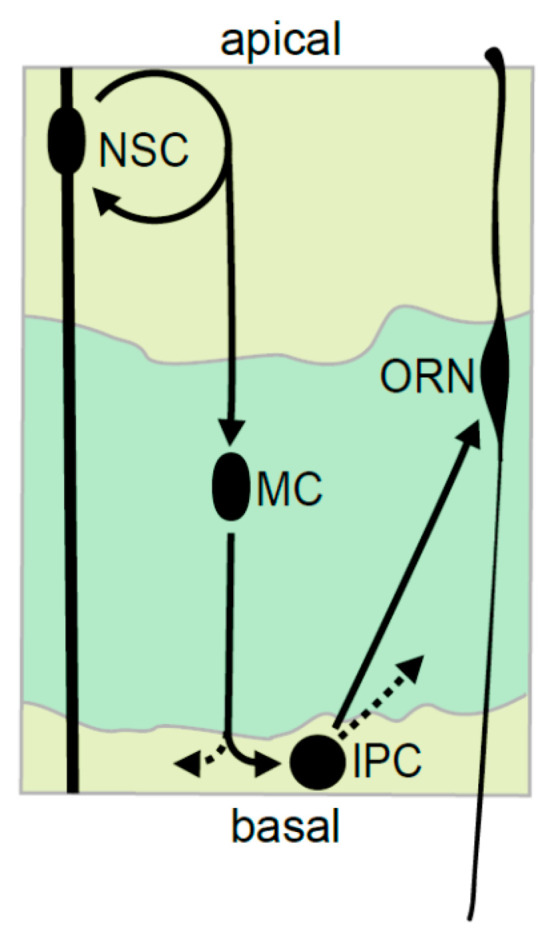
A diagram showing the spatial organization and linear relationships between the neural precursor cell types and the neurons of the mid gestation (E12.5–E15.5) embryonic olfactory epithelium. Abbreviations: NSC—neural stem cell, MC—migrating cell, IPC—intermediate precursor cell, ORN—olfactory receptor neuron.

Developing an experimental model to link neuronal precursor identity and cell cycle phase requires a lineage which contains the principal neural precursor cell types, and in which the spatial position of a cell clearly correlates with its identity. Based on these criteria, we used the mid-gestation embryonic OE (Figure 1) [38]. This lineage has Hes1-expressing apical radial glia (NSCs) [33,35,39]. These NSCs give rise to a small population of Ascl1-positive migrating cells that divide at low frequency when they arrive at the basal surface [32,40,41]. The basal surface of the OE is the site where the IPCs reside (also called basal precursors). They are Neurog1-positive, divide at low frequency, and exit the cell cycle to give rise to neurons (ORNs, Olfactory receptor neurons) [33,34,38,42,43] (Figure S1A–I). Importantly, we show that each cell type is spatially distinct in the tissue: the extended cell bodies of the NSCs form a pseudostratified apical layer, the IPCs are rounded and basal, and the ORNs are sandwiched between these two layers (Figure 1 and Figure S1B,C) [38,39]. We confirmed the markers and spatial organization of the different precursor populations (Figure S1F–H), and in particular, that these precursor populations are marked by the multipotency factor Sox2 [44], and can be defined by the intersection of Sox2 expression and spatial position (Figure S1A–D).

**Figure 2:**
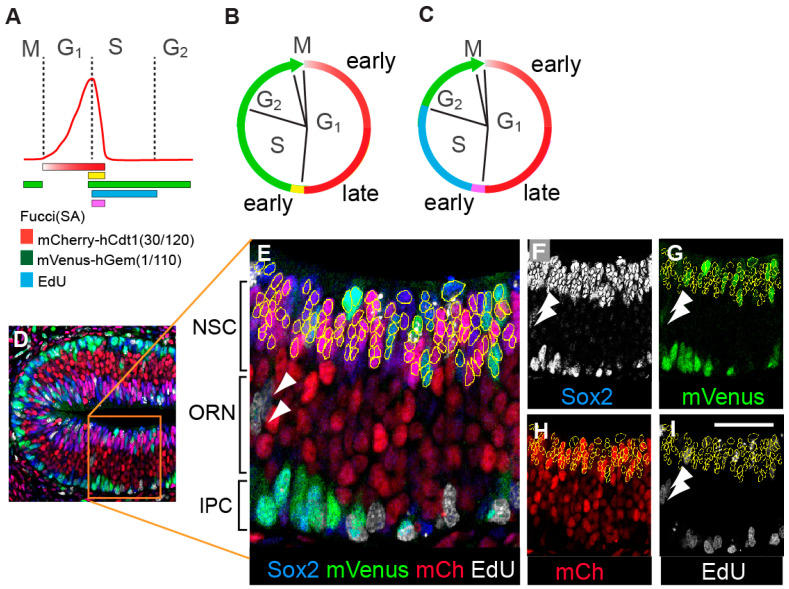
Multiprobe labelling strategies to reveal the cell cycle phases of neural precursors in situ during the elaboration of a neural lineage. (**A**) An illustration of how the relative intensities of Red FP-hCdt1 (30/120) vary during a single cell cycle (adapted from [29]). The bars below the diagram show how cell cycle phases are marked with combinations of the different probes used in this study. (**B**) An illustration of the outcome for dissecting cell cycle phases with the common combination of a Red FP-hCdt1 (30/120) and a Green FP-hGem (1/110). (**C**) An illustration of the outcome for dissecting cell cycle phases when a Red FP-hCdt1 (30/120) and a Green FP-hGem (1/110) are combined with a short pulse of EdU labelling. In this case, S and G2/M phases are now separated. In (**B**,**C**), for color codes see panel (**A**). (**D**,**E**) Multiprobe labelling of E13.5OE using probes shown individually in panels (**F**–**I**). Arrowheads mark migrating cells. Labelling to detect (**F**) anti-Sox2, (**G**) anti-GFP to detect mVenus-hGem (1/110), (**H**) anti-mCherry (mCh) to detect mCherry-hCdt1 (30/120), (**I**) Click-iT to detect EdU. Scale bar = 50 µm.

In parallel, we adapted the use of FUCCI(SA) transgenic constructs [mCherry-hCdt1 (30/120) and mVenus-hGem (1/110)] [28,29,36,45,46] to examine cell cycle phase organization of neural lineage cell types in situ (Figure 2A–C). We added a 30 min pulse of in utero EdU labelling for the detection of all cells in S phase (Figure 2A,C). A lag in the degradation of the G1 phase marker mCherry-hCdt1 (30/120) at the onset of S phase [29,47] means cells co-labelled with EdU and mCherry-hCdt1 (30/120) mark the initiation of S phase. This allowed us to mark definitive G1 exit and S phase entry (Figure 2A,C). When EdU is used in combination with the mVenus-hGem (1/110) S/G2/M phase marker, S phase is subdivided from the rest of the mVenus-hGem (1/110) signal (Figure 2A,C). We optimized multicolor immunohistochemistry (IHC) to allow detection of mCherry-hCdt1 (30/120), mVenus-hGem (1/110), additional cell type specific markers (e.g., Sox2), and Click-iT-based detection of EdU (Figure 2D–I).

### 3.2. Cell Cycle Phase Distribution of OE NSCs and IPCs

**Figure 3:**
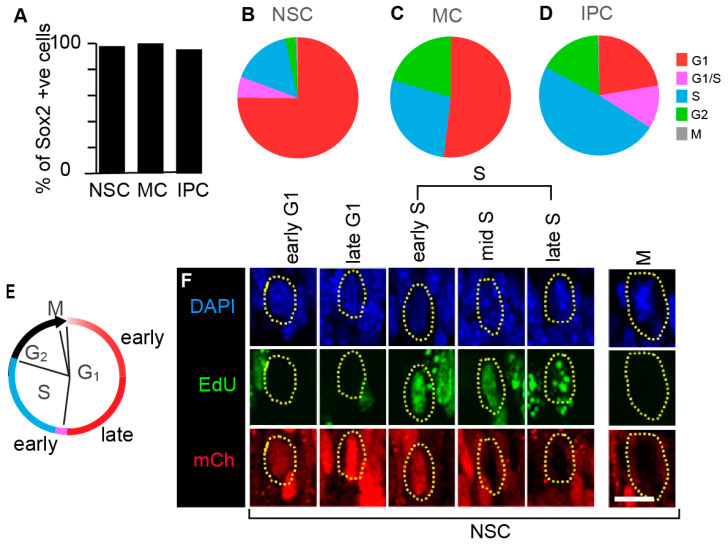
Cell cycle phase distributions in the populations of the three neural precursor cell types of the OE. (**A**) We counted Sox2 positive cells in each position, then quantified the percentage of these cells that were also co-labelled with one or more probes detecting EdU, mCherry-hCdt1 (30/120), and mVenus-hGem (1/110). NSCs—apical position (n = 859). MCs—between the apical and basal domains and not touching either domain (n = 54). IPCs—basal position (n = 324). (**B**–**D**) The cell cycle phase distributions in the populations. (**B**) NSCs are predominantly in G1 (n = 841). (**C**) MCs are intermediate between NSCs and IPCs (there were no G1/S cells detected, n = 54). (**D**) IPCs have many cells in S (n = 324). (**E**) It is possible to distinguish G1 and S phases in the precursor populations by a combination of mCherry-hCdt1 (30/120) and EdU injection. The color code is as in panels (**B**–**D**). (**F**) Labelling of NSCs in different cell cycle phases by a combination of mCherry-hCdt1 (30/120) and EdU. With this IHC protocol, we can clearly distinguish the G1 phase and S phase in the precursors in situ and define the initiation of S phase (labelled as ‘early S’ in this panel and in subsequent figures). Scale bar = 10 µm.

Using the combination of mCherry-hCdt1 (30/120), mVenus-hGem (1/110) and EdU, along with Sox2, we found that 98% of all NSCs, 100% of all MCs and 95% of all IPCs expressed one or a combination of these cell cycle phase markers (Figure 3A).

We then examined the cell cycle phase composition of these different precursor populations (Figure 3B–D). We found that the OE NSC population is primarily in G1 (75%), and that these NSCs have an overall cell cycle phase distribution similar to that previously reported for cultured mouse embryonic cortical NSCs [45] (Figure 3B). However, we found different cell cycle phase organizations for the other populations (Figure 3C,D). Surprisingly, the OE IPC population mainly contains cells in S phase (61%) (Figure 3D). This finding is in strong contrast to previous studies of basal precursor cell populations in the developing mouse, ferret, and macaque cortices that show increases in G1 or reductions in S phase [12,48,49]. This result emphasizes the importance of measuring the cell cycle phase characteristics of each precursor type under study.

In this protocol using optimized IHC, mCherry-hCdt1 (30/120) is detected in the precursor cell populations throughout G1 (Figure 3A). We see 98–100% labelling of NSCs and MCs. For IPCs, 5% are colorless. These colorless cells are likely to be those that have just exited mitosis; as expected [28,29], we did not detect mCherry-hCdt1 (30/120) in any M phase cells (NSCs n = 33; IPCs n = 19). We suggest a higher proportion of the G1 population in IPCs are in this post-division period. As such, a small fraction of G1 cells may be excluded from the IPC G1 population we report; measuring DNA content by DAPI might be a way to further define the identity of colorless IPCs in future studies.

Critically, with this protocol we can now clearly distinguish G1 phase and S phase in the precursor populations in situ without using mVenus-hGem (1/110) (Figure 3E,F). Overall, these findings mean that we could now extend the protocol to carry out experimental analyses of the relationship between additional factors and cell cycle phase.

### 3.3. Differential Organization of Cell Cycle Phase-Linked p27^KIP1^ Levels in Neural Precursor Types

**Figure 4:**
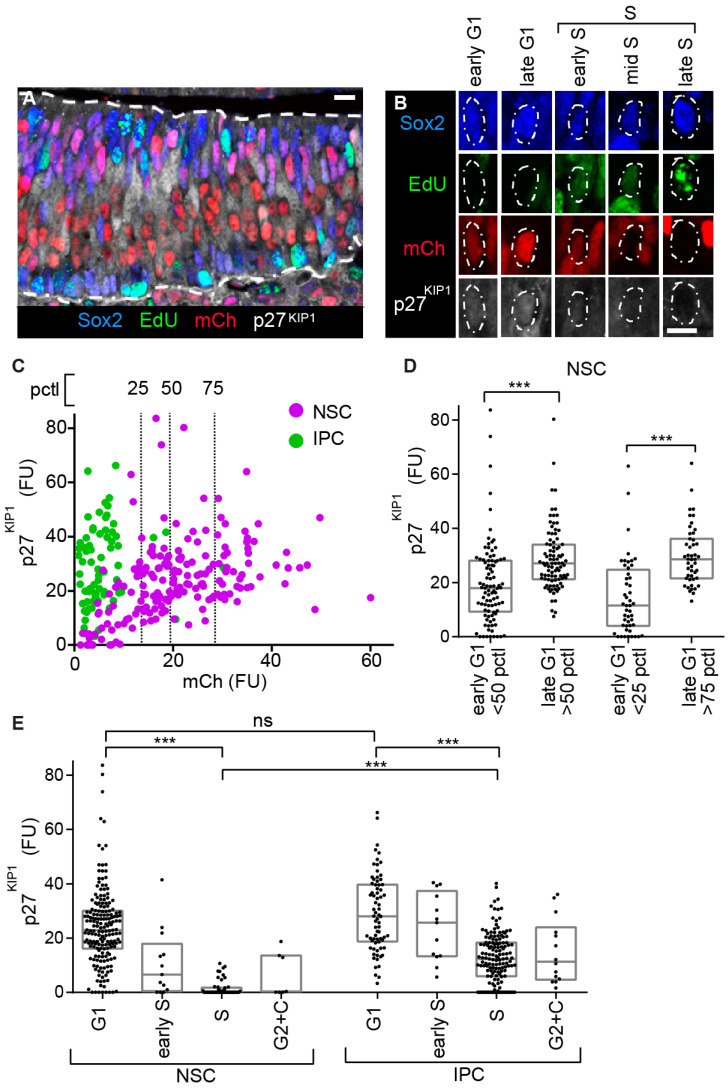
Examination of p27^KIP1^ expression by precursor type and cell cycle phase. (**A**) Labelling of E13.5 OE with a combination of mCherry-hCdt1 (30/120), a 30 min pulse of intraperitoneal EdU injection, anti-Sox2, and anti-p27^KIP1^. (**B**) Division of NSC G1 and S phases using the labelling combination shown in panel (**A**). (**C**) A comparison between mCherry-hCdt1 (30/120) and p27^KIP1^ levels (the mCherry-hCdt1 (30/120)-positive, EdU-negative cells were examined). The vertical dashed lines show the 25th, median, and 75th percentiles (pctl) of mCherry-hCdt1 (30/120) levels in the NSC population. Pearson correlation analysis of mCherry-hCdt1 and p27^KIP1^ levels in the NSCs: r = 0.40, *p* < 0.0001 (NSCs n = 185, IPCs n = 72). (**D**) Box plots showing comparison of p27^KIP1^ levels for early and late phases as defined by the staining levels of mCherry-hCdt1 (30/120). We compare the population of cells above and with that below the 50th percentile (pctl) for mCherry-hCdt1 (30/120) levels (n = 92, 93). Similarly, above and below the 75th and 25th pctls, respectively (n = 47, 48). FU—average intensity in fluorescence units. (**E**) Box plots showing p27^KIP1^ levels by cell cycle phase for NSCs and IPCs (n = 185, 13, 50, 7, 72, 13, 149, 14). In plotting these graphs, we combine cells in G2 with the small percentage of cells (2% in NSCs and 5% in IPCs) that are an undefined colorless state (labelled as C). (**D**,**E**) Kruskal–Wallis test with Dunn′s multiple comparisons. ns (not significant), * (*p* ≤ 0.05), ** (*p* ≤ 0.01), *** (*p* ≤ 0.001); scale bars = 10 µm

The CDKi p27^KIP1^ is a key regulator of neuronal lineage progression; nevertheless, its expression and regulation have remained unclear [11,13,14,18,20,22,24,25,26,27,50]. In cycling cells, p27^KIP1^ protein levels increase during the progression of G1, then drop sharply at the onset of S phase [1,21]. To visualize whether this canonical organization occurs in the different neuronal precursor types, we labelled the OE to detect mCherry-hCdt1 (30/120), EdU, Sox2, and p27^KIP1^ (Figure 4A,B). In all these experiments, we specifically measured nuclear p27^KIP1^ levels [1,21] (as expected, p27^KIP1^ is both nuclear and cytoplasmic in the post mitotic nascent neurons [13]).

In proliferating cells, the relatively slow rate at which a FP-hCdt1 (30/120) protein matures means this marker is active at a weak level at the initiation of G1 phase, then increases as the cell proceeds through G1. This useful property (relative to alternative FUCCI versions [29,47]) allows the investigator to distinguish between cells in early and late G1 phase and has been used to subdivide the G1 of cultured cells by FACS [28,29,45,46,51,52]. We therefore examined the relationship between mCherry-hCdt1 (30/120) and nuclear p27^KIP1^ levels in the NSCs, as these have extensive G1. We saw a positive correlation (Figure 4C). Then, we further quantified the specific late G1 phase enrichment of p27^KIP1^ expression in the NSCs after subdividing them into early and late G1. This revealed a 46% upregulation in mean levels when dividing the population at the median, and a 105% upregulation when comparing above and below the 75th and 25th pctls (Figure 4C,D and Figure S2A).

To investigate the attributes of the different neural precursor populations, we assayed p27^KIP1^ levels across cell cycle phases. As expected [1,21], in both NSCs and IPCs, p27^KIP1^ levels are higher in G1 than S phase (Figure 4E). Nevertheless, while p27^KIP1^ levels in NSCs drop to low or undetectable in S phase, they do not undergo a similar drop in IPCs (Figure 4E and Figure S2B). In NSCs the p27^KIP1^ mean level drops 93% when moving from G1 to S, while in IPCs this difference is 57%. Moreover, the mean p27^KIP1^ level in S phase is 88% lower in NSCs relative to IPCs. These data reveal a differential organization of p27^KIP1^ with respect to cell cycle progression between the NSCs and IPCs.

### 3.4. Hes1 in NSCs Regulates p27^KIP1^ Levels in IPCs

**Figure 5:**
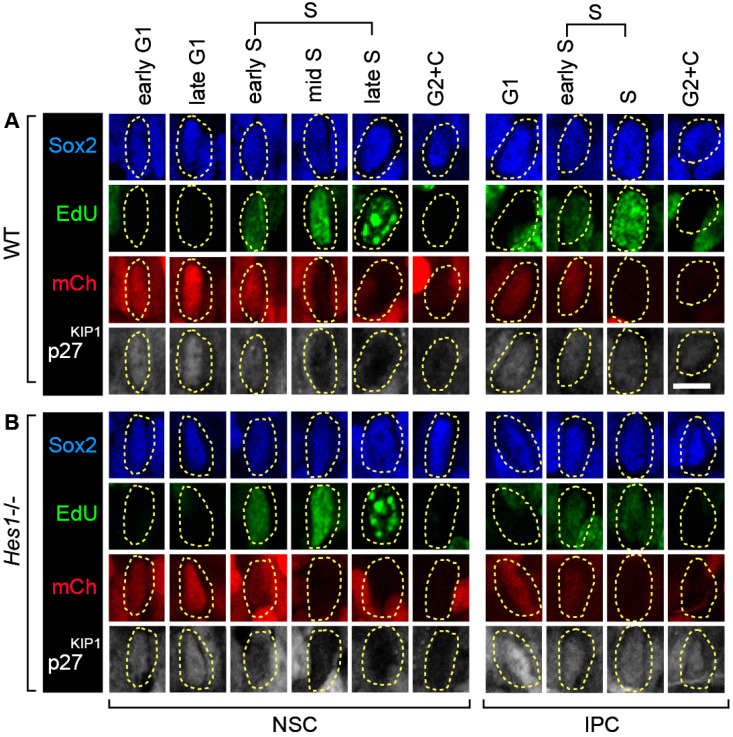
Multiprobe immunostaining of OE NSCs and IPCs using antibodies to detect Sox2, mCherry-hCdt1 (30/120) (abbreviation: mCh), and p27^KIP1^, and in addition with Click-iT to detect EdU. (**A**) Wildtype (WT), (**B**) *Hes1-/-*. p27^KIP1^ levels are upregulated in *Hes1-/-* IPCs, but not in NSCs. Scale bar = 10 µm.

**Figure 6:**
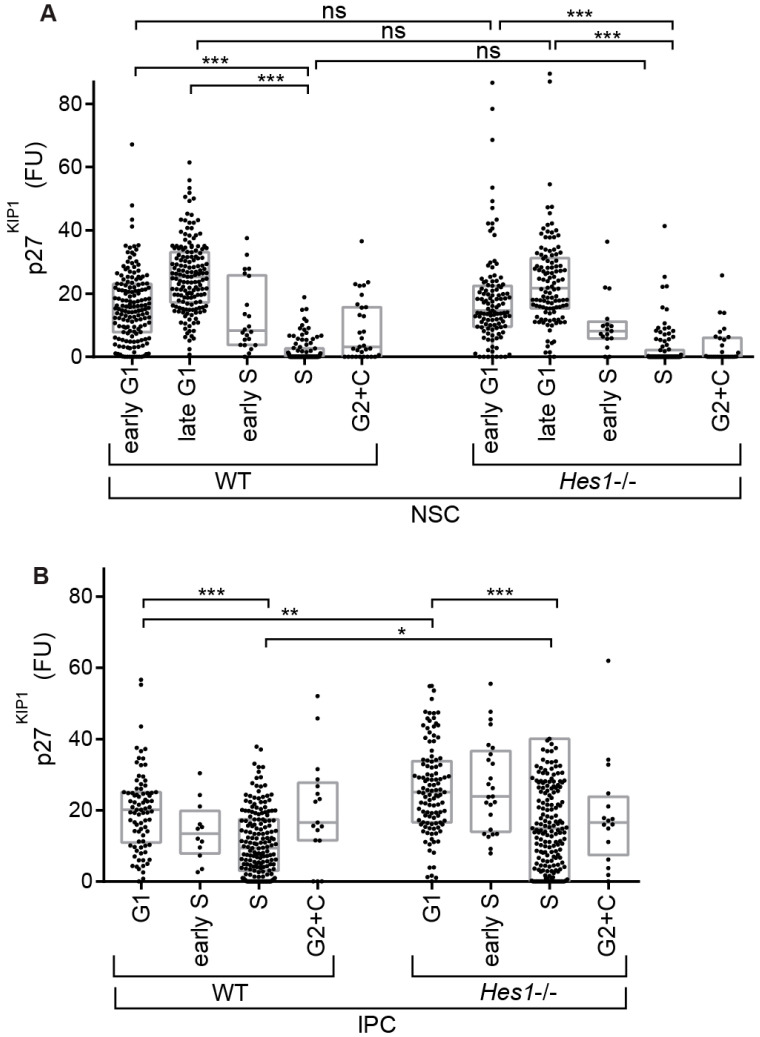
Box plots comparing p27^KIP1^ levels between WT and *Hes1-/-.* (**A**) NSCs (n = 159, 159, 22, 98, 31, 115, 114, 17, 95, 29); (**B**) IPCs (n = 82, 12, 181, 17, 113, 25, 175, 16). These data show p27^KIP1^ levels are upregulated in *Hes1-/-* IPCs, but not in NSCs. FU–average intensity in fluorescence units. Krustal-Wallis tests with Dunn′s multiple comparisons. ns (not significant), * (*p* ≤ 0.05), ** (*p* ≤ 0.01), *** (*p* ≤ 0.001).

Given our finding that there is differential organization of p27^KIP1^ levels between the different precursor types during the elaboration of the lineage, we further investigated the regulatory relationship between Hes1 and p27^KIP1^ in these cells. We crossed R26p-Fucci2 [36] into a *Hes1* knockout background [37]. Then, we confirmed that loss of *Hes1* activity causes a reduction in NSC number (Figure S2C). Next, we examined p27^KIP1^ levels across cell cycle phases in each of the different neural precursor populations (Figure 5A,B, Figure 6A,B and Figure S2D).

We found no change in p27^KIP1^ levels in NSCs, suggesting that p27^KIP1^ levels are not directly regulated by Hes1 in these cells (Figure 6A). This is important, because previous studies have predicted a direct repression of p27^KIP1^ expression by Hes1 [22,24,25,26]. We also did not detect clear changes in p27^KIP1^ levels between WT versus *Hes1*-/- MCs. We note that p27^KIP1^ expression is not lost in S phase in these cells, suggesting that increases in S phase p27^KIP1^ levels may begin as the cells are in the migrating stage (Figure S2D).

Surprisingly, the result of loss of *Hes1* was detected in IPCs. In the IPCs, p27^KIP1^ levels were still higher in G1 than S phase in the *Hes1-/-* mutant, but there was now a further IPC-specific regulatory outcome added on top. Loss of *Hes1* caused upregulation of p27^KIP1^ levels, both in G1 and S phases (by 31% and 38%, respectively) (Figure 6B). In keeping with this, we noted a trend in the cell cycle phase organization towards more cells in G1 phase (Figure S2E). Importantly, these data indicate that p27^KIP1^ is regulated by Hes1 during neurogenesis. However, remarkably, this regulation is indirect; the output is in the IPCs rather than the NSCs. In this way, Hes1 in NSCs controls not only the switch between self-renewal and differentiation in these cells [23,31], but also cell cycle regulator organization in precursors downstream in the lineage.

## 4. Discussion

We can now visualize the relationships between neural precursor type, cell cycle phase, and candidate factor expression. We can do this for each individual cell in situ during OE lineage progression. The protocol can also be adapted to examine development and organization of other tissues.

In this study, we used the embryonic OE neural lineage as a model with powerful advantages to examine links between cell cycle phase and cell fate switches. One clear difference between cortical and other areas (either in simple lissencephalic or complex gyrated brains) is streamlining from multiple basal precursors into a single basal precursor/IPC cell type [2]. Importantly, the NSCs and IPCs are in spatially different domains, and can be unambiguously identified by both marker gene expression and position [38]. Additionally, Hes1 is the only Hes-family factor expressed in the NSCs, and it is exclusive to these cells [31]. Overall, the streamlined OE lineage has the key hallmarks of a Notch-Hes-mediated NSC self-renewal vs. differentiation switch, and a proneural cascade controlling the sequential steps of NSC-IPC-neuron differentiation [31,32,33,34,53,54]—and it also allows clear spatial visualization of these sequential steps in situ [38].

We used this model system to investigate regulation of p27^KIP1^. We find that p27^KIP1^ maintains its canonical G1-phase-linked expression in both the OE NSCs and IPCs, but that superimposed upon this in IPCs, p27^KIP1^ protein does not drop to low levels as the cell transitions through the S phase. Remarkably, we find that global upregulation of p27^KIP1^ levels in the IPCs is regulated by Hes1, which is only expressed in the NSCs. This surprising result rationalizes previously conflicting findings. It is unclear how Hes1 causes this indirect effect on p27^KIP1^ levels. As p27^KIP1^ and proneural proteins can stabilize each other [13,55], we hypothesize that this p27^KIP1^ upregulation might be due to dysregulation of the proneural transcription factor cascade in the lineage when *Hes1* is lost [31]. The new finding that p27^KIP1^ becomes dysregulated in the final precursor stage downstream of regulatory control in the cells at the head of the lineage is important because upregulation of p27^KIP1^ in neural precursors increases the probability that they initiate terminal differentiation [11,13,14,22,50].

Simultaneous readout of precursor identity and cell cycle phase will now enable further key questions to be investigated. To what degree are neuronal precursor cell fate transitions associated with cell cycle phase transitions? Seminal transplantation studies in the ferret cortex showed that early stage cortical precursor cells commit to adopting a specific fate prior to G2 [56]. Moreover, lineage trajectories derived from single cell sequencing studies capture cells in transition between defined cell fates [57], and in recent single cell sequencing studies of primate and human organoids and embryonic cortices, these cells that are in transition between defined precursor fates are also enriched for subsets of cell cycle phase markers [58,59]. Moreover, how does progression through a specific cell cycle phase modulate the differentiation program? In NSCs and IPCs this is not clear, but hints can come from examining other cell types where G1 has been shown to be a specific window that allows interpretation of signal receipt. For example, retinoic acid induces embryonal carcinoma cells to differentiate, but only when this signal is received during G1 [60]. Additionally, when ESCs respond to TGFβ, in early G1, the Smad2/3 transcriptional effector complex binds endoderm genes initiating endodermal differentiation; however, in late G1, Smad2/3 are no longer able to access their targets and the same signal does not activate this program [46]. Surprisingly, recent data also indicate that, rather than the phase length parameters of any individual cell, in ESC cultures the amount of variation of cellular G1 lengths within the population is the best predictor of cell fate decisions [51]. This is an intriguing result that serves to emphasize a further key issue—how do the different precursor cell types of the lineage interact at a systems level?

Overall, the field must develop new assays to look at the organization and interaction of precursors in situ. We suggest the model we describe here provides one important tool towards achieving this goal.

## Supplementary Materials

The following are available online at https://www.mdpi.com/2073-4409/9/9/2112/s1, Figures S1–S2.
