# Supplementary material for: Visualizing Cell Cycle Phase Organization and Control During Neural Lineage Elaboration
Source: Cells. 2020 Sep 17;9(9):2112. doi: 10.3390/cells9092112 (PMC7565168; doi:10.3390/cells9092112)
Supplement: Supplementary file 1 [file cells-09-02112-s001.pdf]

Supplementary Materials: Visualizing Cell Cycle Phase Organization and Control During Neural Lineage Elaboration.

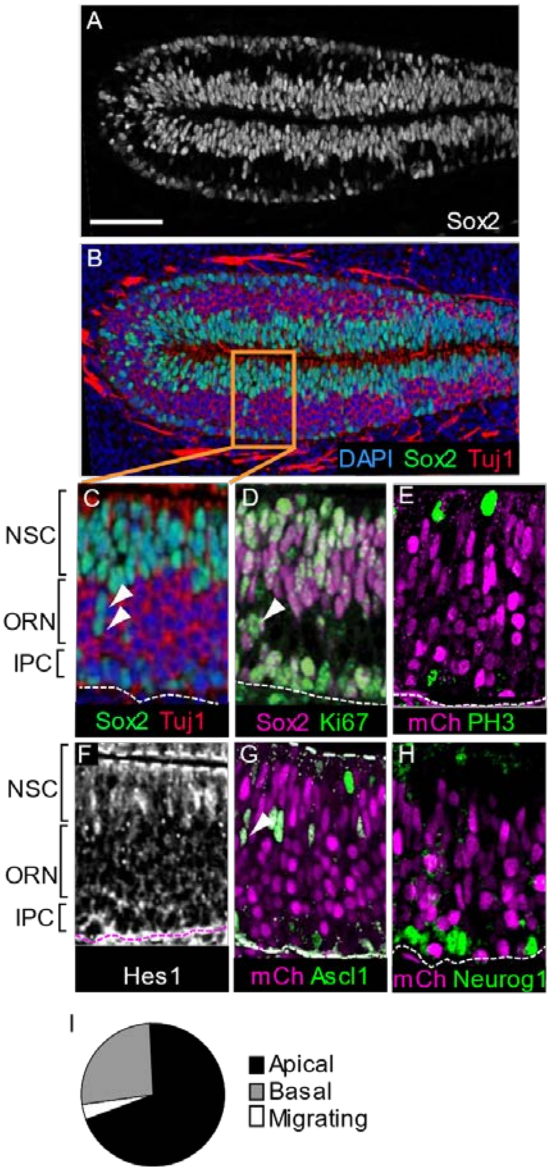

Figure S1. Immunohistochemical labelling showing the spatial organization of cell types in the mid gestation (E13.5) OE (**A–C**) Sox2 marks all neural progenitor populations; Tuj1 (β3Tubulin) marks the neurons. (**D**) The Sox2 positive cells express the cellular proliferation marker Ki67. Migrating cells are marked with an arrowhead. (**E**) Mitosis (shown by anti Phospho-Histone H3 labelling) occurs in the apical and basal precursor domains. (**F–G**) The different precursor populations have different molecular identities; they express different transcription factor components of a proneural cascade. (**F**) Hes1 is expressed in the NSCs (apical domain). (**G**) Ascl1 marks cells in both precursor domains as well as the migrating cells. (**H**) Neurog1 is expressed in the IPCs (basal domain). (**I**) A pie chart showing the relative numbers of each cell type (n = 1226). Scale bar = 100μm.

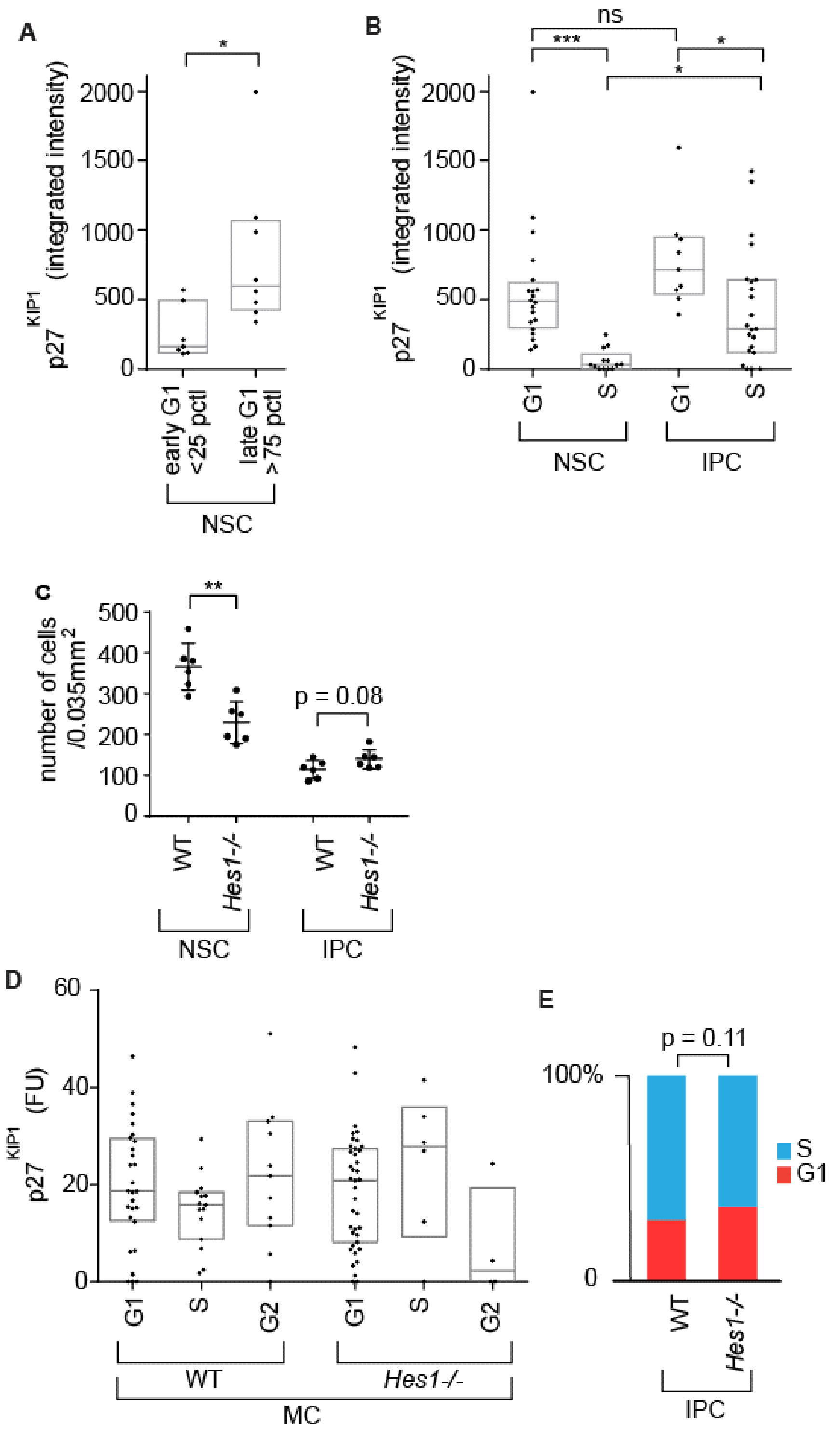

Figure S2. (**A**, **B**) Box plots showing p27^{KIP1} levels per cell cycle stage. The data shows integrated intensity measured from cells where a confocal section is through the nucleus at maximum area (**A**) (n =7, 8) (**B**) (n =20, 13, 9, 23). Krustal-Wallis tests with Dunn's multiple comparisons. (**C**) Scatter plots (mean +/- SD) show the number of precursors in WT () versus *Hes1-/-* OE. Students two tail t-tests (n = 6, 6, 6, 6) (**C**) Box plots showing p27^{KIP1} levels per cell cycle stage and comparing WT and *Hes1-/-* in the MCs (n = 28, 15, 11, 39, 6, 4). FU - average intensity in fluorescence units. We were unable to detect significant changes between any population (Krustal-Wallis with Dunn's multiple comparisons). These data suggest that p27^{KIP1} levels are not dropping to low levels in S phase in the MCs. (**D**) The relative proportion of G1 and S phase (all EdU-positive) cells in the IPC in WT and *Hes1-/-* backgrounds. Chi-squared test (n = 275, 313). ns (not significant), * ($p \leq 0.05$), ** ($p \leq 0.01$), *** ($p \leq 0.001$).
